# Surgical versus non-surgical treatment of humeral SHAFT fractures compared by a patient-reported outcome: the Scandinavian Humeral diAphyseal Fracture Trial (SHAFT)—a study protocol for a pragmatic randomized controlled trial

**DOI:** 10.1186/s13063-022-06317-6

**Published:** 2022-06-02

**Authors:** Dennis Karimi, Stig Brorson, Kaare S. Midtgaard, Tore Fjalestad, Aksel Paulsen, Per Olerud, Carl Ekholm, Olof Wolf, Bjarke Viberg, SHAFT Collaborators, Katharina Stohlmann, Bamo Jalal, Christian Cavallius, Esben S. Pedersen, Frede Frihagen, Frederik Stensbirk, Henrik Illerström, Jens Knak, Anne Marie Nyholm, Jesper Schønnemann, Joakim Jensen, Jonas Sundkvist, Mads Vinding, Peter M. Siesing, Srdjan Zivanovic, Søren Kring

**Affiliations:** 1 Department of Orthopedic Surgery, Kolding Hospital, Kolding, Denmark; 2 Department of Orthopedic Surgery, Centre for Evidence-Based Orthopaedics, Zealand University Hospital, Køge, Denmark; 3 Department of Clinical Medicine, University of Copenhagen, Copenhagen, Denmark; 4 Department of Orthopedic Surgery, Oslo University Hospital, Oslo, Norway; 5 Department of Orthopedic Surgery, Stavanger University Hospital, Stavanger, Norway; 6 Department of Orthopedic Surgery, Stockholm South General Hospital, Stockholm, Sweden; 7 Department of Orthopedic Surgery, Sahlgrenska University Hospital, Gothenburg, Sweden; 8 Department of Surgical Sciences, Section of Orthopedics, Uppsala University, Uppsala, Sweden

**Keywords:** Humeral shaft fracture, Diaphyseal fracture, Treatment, Surgical fixation, Non-surgical, Delayed union, Patient-reported outcomes, Randomized Controlled Trial

## Abstract

**Background:**

The outcome of non-surgical treatment is generally good, but the treatment course can be long and painful with approximately a quarter of the patients acquiring a nonunion. Both surgical and non-surgical treatment can have disabling consequences such as nerve injury, infection, and nonunion. The purpose of the study is to compare patient-reported outcomes after surgical and non-surgical treatment for humeral shaft fractures.

**Methods:**

A pragmatic randomized controlled trial (RCT) is planned with two study groups (SHAFT-Young and SHAFT-Elderly). A total of 287 eligible acute humeral shaft fractures are scheduled to be recruited and randomly allocated to surgical or non-surgical treatment with the option of early crossover due to delayed union. The surgical method within the allocation is decided by the surgeon. The primary outcome is the Disability of Arm, Shoulder, and Hand (DASH) score at 52 weeks, and is assessor blinded. The secondary outcomes are DASH score, EQ-5D-5L, pain assessed by visual analog score, Constant-Murley score including elbow range of motion, and anchor questions collected at all timepoints throughout the trial. All complications will be reported including; infection, nerve or vascular injury, surgical revisions (implant malpositioning, hardware failure, aseptic loosening, and peri-implant fracture), major adverse cardiovascular events, and mortality.

**Discussion:**

The SHAFT trial is a pragmatic multicenter RCT, that will compare the effectiveness of the main strategies in humeral shaft fracture treatment. This will include a variety of fracture morphologies, while taking the dilemmas within the population into account by splitting the population by age and providing the orthopedic society with an interval for early crossover surgery.

**Trial registration:**

Clinicaltrials.govNCT04574336. Registered on 5 October 2020.

**Supplementary Information:**

The online version contains supplementary material available at 10.1186/s13063-022-06317-6.

## Background and rationale

The incidence of humeral shaft fracture is between 13.5 and 20 per 100.000 annually [1, 2]. It is projected that the incidence of humeral shaft fractures will increase due to changing demographics [1]. The fracture demography follows a typical bimodal pattern with young adults injured in sports, vehicular road-traffic accidents, and other high energy traumas and the elderly injured with simple falls, respectively [2]. Considering this demographic difference, the importance of age in terms of patient expectations, upper limb function, and patient-reported outcome measures (PROM) in humeral shaft fracture treatment is not well understood. The most utilized PROM of humeral shaft fractures across age is the Disability of Arm, Shoulder, and Hand (DASH) [3]. Normative data from the general population shows inferior DASH scores with increasing age and suggests that DASH scores of age groups should not be compared [4, 5].

One of the main challenges with humeral shaft fractures is the choice between surgical and non-surgical treatment. Primary surgical treatment provides consistent high union rates, but patients are exposed to the risk of complications such as infection, iatrogenic radial nerve lesion, and rotator cuff injury as well as shoulder impingement [6, 7]. In contrast, union rates from non-surgical treatment can vary from 75 to 100% [8–12]. If nonunion occurs, it is not uncommon for patients to go through a prolonged treatment course up to 8 months before an additional procedure is offered [12, 13]. Although delayed surgical fixation leads to high union rates [14], the PROMs are inferior compared to primary fixation [13, 15, 16]. These observations may suggest that the prolonged course for patients with union problems is unfavorable. However, the challenge is to identify the patients that will benefit from early fixation. A way of determining early onset of union problems in a young cohort is by gently testing the fracture site at 6 weeks [17] and later for the elderly [18]. In 2020, three systematic reviews with meta-analyses [19–21] were published comparing the surgical and non-surgical treatment of humeral shaft fractures. All studies concluded the need for future prospective randomized controlled trials (RCTs) to complement the current literature in determining the optimal management of these fractures. None of the systematic reviews [19–21] included the RCTs from Finland or Iran [13, 22].

To our knowledge, four RCTs have been completed and all trials compared plate osteosynthesis to non-surgical treatment by DASH score and without distinction of age [13, 22–24]. Four RCT protocols [25–28] are registered in clinicaltrials.gov, WHO and ISRCTN registry. Two RCT protocols are comparing plate osteosynthesis to non-surgical treatment and the last two RCT protocols are comparing plate and nails to non-surgical treatment. Adults of all ages are included, except in one RCT protocol [27] that has an upper age limit at 65 years. Furthermore, in one already finished trial, the trial design had to be adjusted from an RCT to a prospective non-randomized comparative trial due to treatment allocation problems as there was a strong physician preference towards the surgical treatment option [29]. A Cochrane review could not demonstrate any difference in union rate between different surgical procedures (intramedullary nail and plate osteosynthesis) [30].

This emphasizes the need for a pragmatic approach of interventions by including usual care of surgical treatment, considering the influence of age as well as accepting early secondary surgery as a part of treatment to improve disability and function after 12 months.

The SHAFT protocol conforms with the Standard Protocol Items: Recommendations for Interventional Trials (SPIRIT) [31, 32].

## Methods: Participants, interventions, and outcomes

### Objectives

To compare surgical fixation of humeral shaft fracture to non-surgical treatment with early identification and treatment of delayed union by a patient-reported outcome after 52 weeks.

The trial population is divided into two age groups due to the changes in DASH score by age [4]. The definition of delayed union differs in the young and elderly population to consider dissimilarity in bone healing rates:

1. SHAFT-Y for the young with an age cut-off of 18 to 64 years. The early identification and treatment of delayed union is set to 6 to 12 weeks
2. SHAFT-E for the elder with an age cut-off +65 years. The early identification and treatment of delayed union is set to 12 to 26 weeks

### Trial design

**Fig. 1:**
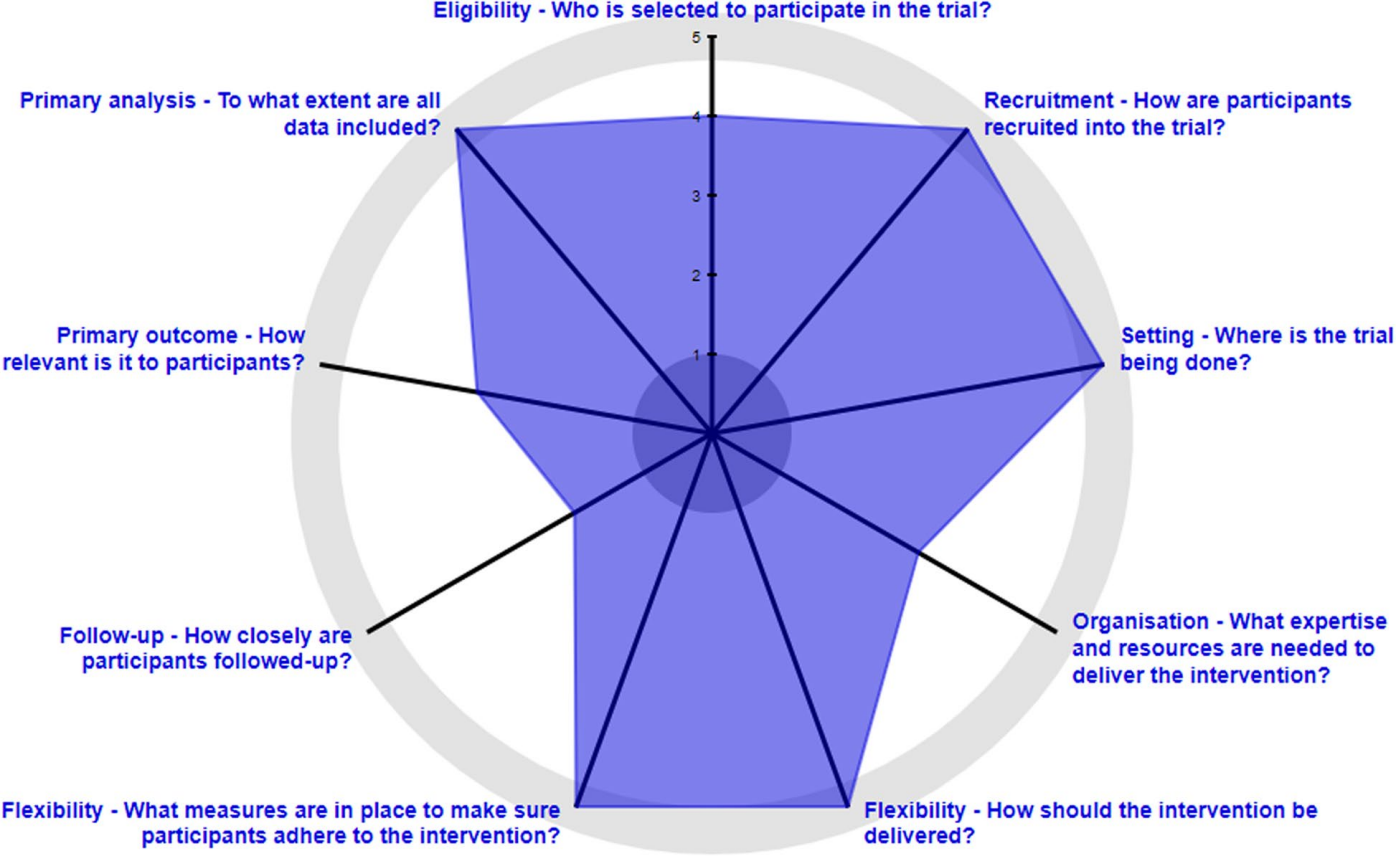
Assessment of the pragmatic design using the PRECIS-2

A pragmatic multicenter, randomized, controlled, outcome assessor-blinded, clinical superiority trials. The steering committee which consist of trauma- and shoulder-elbow surgeons have assessed the pragmatic design using PRECIS-2 [33] which yielded 37 points out of the possible 45 points (Fig. 1). An explanation for each domain score is provided in Appendix 1. This study furthermore collaborates with the NORCRIN Work Package 13 network.

### Study setting

Sites from Denmark, Sweden, and Norway have been recruited and span from academic level I to level III trauma centers. The following is the current list of recruiting hospitals: Kolding Hospital; Hvidovre University Hospital of Copenhagen; Zealand University Hospital; Slagelse Hospital; Holbæk Hospital; New North Zealand University Hospital of Copenhagen, Odense University Hospital; Hospital of Southern Denmark; Aalborg University Hospital; Aarhus University Hospital; Herlev-Gentofte University Hospital of Copenhagen, Viborg Regional Hospital, Hospital South West Jutland, Oslo University Hospital; Stavanger University Hospital; Østfold Hospital Trust; Sahlgrenska University Hospital; Uppsala University Hospital; Umeå University Hospital; and Stockholm South General Hospital.

### Material

**Fig. 2:**
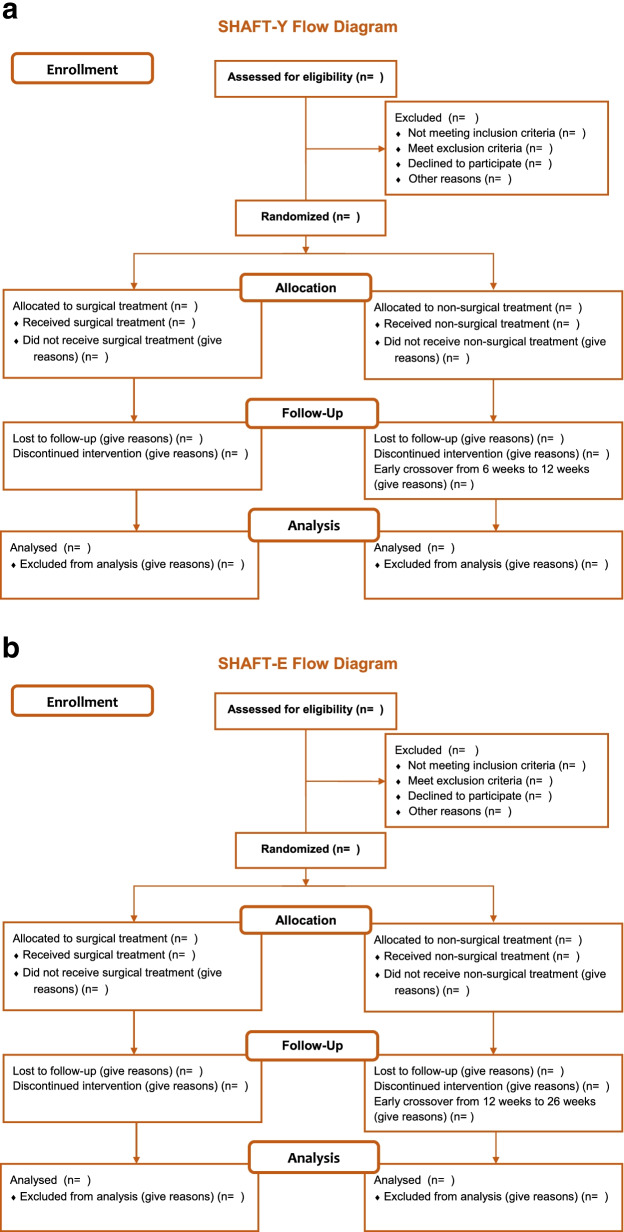
**a** Trial flow for SHAFT-Y. **b** Trial flow for SHAFT-E

**Fig. 3:**
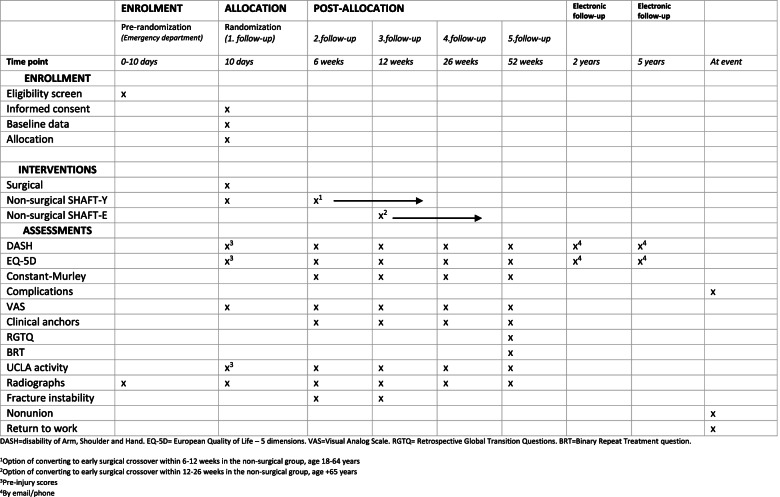
Timeline and overview of enrollment, interventions, and assessments for SHAFT

Two hundred eighty-seven patients (*n*=163 for SHAFT-Y, *n*=124 for SHAFT-E) with a humeral shaft fracture will be equally randomized to surgical treatment or non-surgical treatment in each group. The overall trial flow and timeline of data collection is outlined in Figs. 2a, b and 3.

### Inclusion criteria

1. Fracture types 12A-C (OTA/AO classification)

- Includes minimal displaced extra-articular fracture extensions to the proximal humerus (less than a 1-cm or 45° angulation) [34]

2. Treatment within 14 days from trauma
3. Age 18–64 years for SHAFT-Y and ≥65 years for SHAFT-E
4. Patients must understand the information given and be able to read and speak Danish, Swedish or Norwegian to complete the study paperwork

**Fig. 4:**
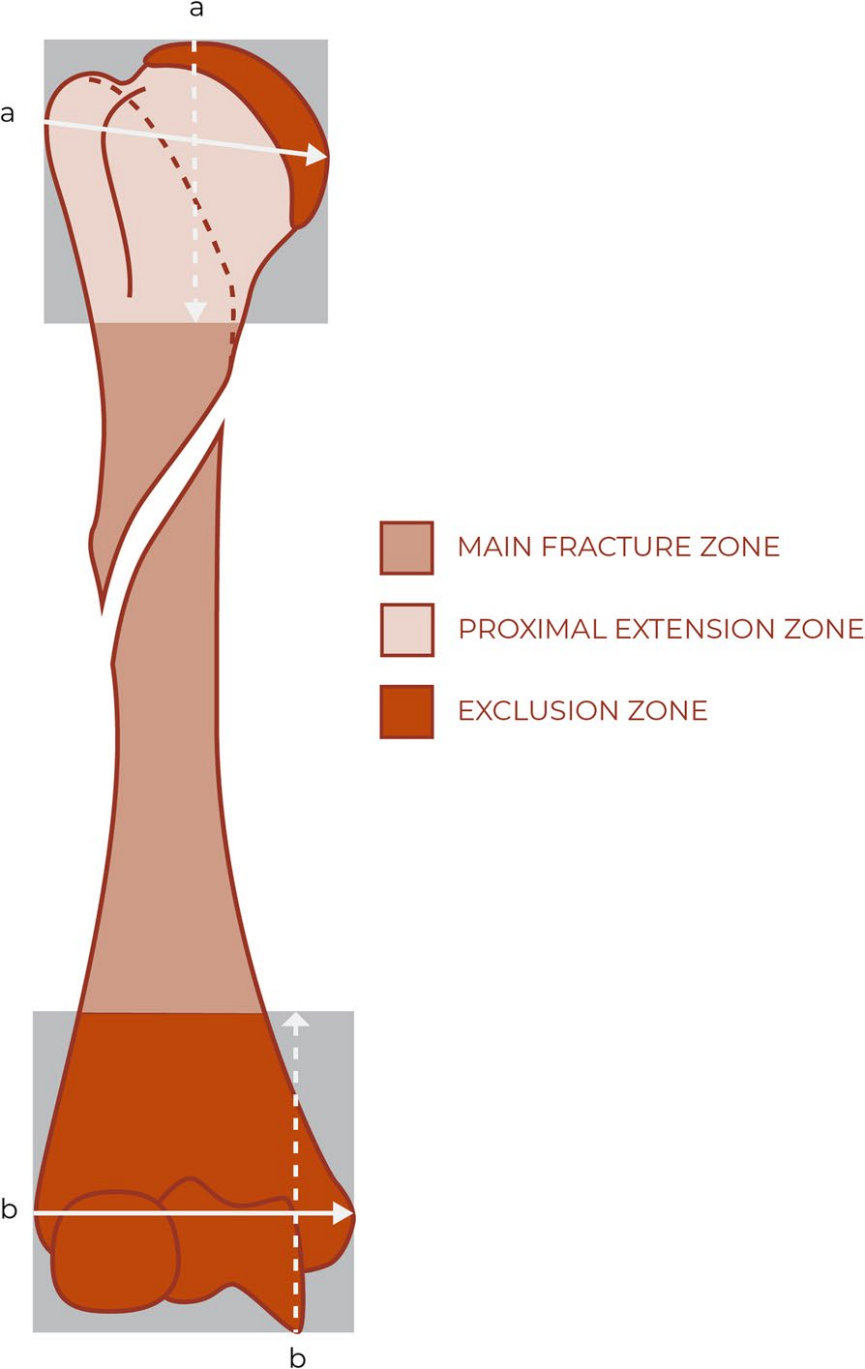
Illustration of the main fracture zone, the extension zone, and the exclusion zones. All fractures must involve the main fracture zone. Fractures can extend to the proximal extension zone, if minimal displaced within the extension zone. Fractures cannot involve the exclusion zones or be displaced within the extension zone. The proximal square is obtained by measuring the widest part of the segment, then measuring the same length from the apex of the proximal segment through the axis of the proximal humerus, this endpoint separates the proximal humerus from diaphysis. The distal square is obtained by measuring the widest part of the segment, then measuring the same length from the most distal part of the trochlea through the axis of the distal humerus, this endpoint separates the distal humerus from the diaphysis

All fracture extensions involving the distal humerus and displaced fracture extensions involving the proximal humerus will not be included. Isolated fractures to the proximal or the distal end of the humerus are not eligible for screening. The proximal and distal end segments of the humerus are defined by squares of which the sides are the widest length of the epiphysis/metaphysis in question on the anterior-posterior view [35] (Fig. 4).

### Exclusion criteria

1. Inability to give informed consent
2. Undisplaced shaft fracture (less than a cortex-wide displacement in all radiographic planes)
3. Vascular injury in the ipsilateral arm
4. Polytrauma (defined as a trauma with one or more concurrent fractures to the upper extremities or other trauma absolute indications for surgical intervention)
5. Pathological fracture
6. Open fracture
7. BMI > 40
8. Health conditions preventing either treatment

Primary radial nerve palsy (RNP) is not an exclusion criterion as there have not been shown benefits in recovery time with early exploration [36].

### Recruitment

Patients admitted to the emergency department (ED) in any of the trial sites will be clinically examined and plain radiographs will be obtained to confirm the diagnosis. If the patient fulfills the eligibility criteria, they will be informed of the trial by staff and receive written information with patient participation information and “Researchers rights in a health science research project.” They will be given time to consider and will be scheduled for an appointment with research staff within 10 days. If written consent is obtained at the consultation, randomization will occur immediately after.

Patients will be provided with transport allowance to cover the costs for consultations at 26 weeks and 52 weeks. They further accept telephone calls if they miss follow-up visits to promote participant retention and complete follow-up.

### Interim analyses

We will carry out two interim analyses after trial completion of 25% and 50% of the planned number of included patients, separately for the two age groups. We will use the O’Brien-Fleming rule [37] for determining appropriate significance levels for each analysis, which is determined by time of hypothesis testing (*n*=3) and the overall significance level of *α*=0.05. Under these assumptions, the significance levels are 0.0006 for the first interim analysis, 0.0151 for the second interim analysis, and 0.0471 for the final analysis. Interim analyses will be carried out for both the primary endpoint (DASH) and for serious complications (iatrogenic nerve injury, deep infection, major adverse cardiac events (MACE), other major adverse events, and death) and will be conducted by one-sided tests to determine if the improvement in DASH is significantly above 25 points, respectively if the complication rate is at least 20% higher.

A Data Monitoring Committee (DMC) has been organized to monitor and evaluate the data from the interim analyses. The DMC consists of five independent members including the patient representative (PR), two orthopedic researchers, an orthopedic surgeon, and a nurse.

In the event of one group having zero complications, the significance level cannot be computed by a cChi-square test, and the DMC will have to assess the data due to a statistical variation.

In the event of a patient sustaining several complications, the most severe complication will follow the patient. The complications are ranked in a hierarchy model describing the severity and are ranked from most severe to least severe: death, MACE, deep infection with debridement, other reasons for hospitalization, and iatrogenic nerve injury.

The interim analyses will be conducted by a biostatistician, blinded to the treatment allocation. Data will then be presented to the DMC, in means and proportions and be accompanied with confidence intervals. The DMC will have access to the data unblinded, and if at least one of those two conditions is fulfilled or a statistical variation has occurred at an interim analysis the DMC will be asked to investigate the results in detail and present their recommendation to continue or stop the trial. In the event of disagreement with a split decision (one or more casts a blank vote), the steering committee will be involved in discussing the stopping of the trial.

Furthermore, the steering committee will be monitoring recruitment and drop-out. Any modification in design and recruitment will be registered on ClinicalTrials.gov.

If it is observed that inclusion of patients in the two age groups is too slow, it can be decided to pool the age groups and analyze them as one total group instead. If this decision is made before the first interim analysis, the above significance levels will be used for the total group. If the decision is made between the first and second interim analysis, O’Brien-Fleming significance levels for four analyses will be used, resulting in a significance level of 0.0184 for the second interim analysis and 0.0412 for the final analysis. If the decision is made after the second interim analysis, the O’Brien-Fleming significance level for five analyses will be used, resulting in a significance level of 0.0417 for the final analysis.

### Patient and public involvement

Patients were involved in the planning and development of the study protocol. A series of in-depth semi-structured interviews were conducted with the qualitative purpose of exploring the experiences acquired during the treatment course of a humeral shaft fracture. Beyond the qualitative study aim, a discussion of the most meaningful primary outcome measures was undertaken. All patients completed two questionnaires (DASH and QuickDASH) to solicit their feedback and to determine which PROM they found most appropriate when considering relevance, comprehensibility, comprehensiveness, and length. The interviews further revealed complaints that were discussed with the patients and were subsequently priorities to be implemented as outcome measures of the trial.

One of the patients from the interviews accepted to be involved in the trial as the PR. The PR is included in the steering committee and in the DMC. The study protocol was discussed with the PR in layman’s terms to facilitate a common understanding of the trial and to solicit feedback that could minimize patient burden and risk of missing data, as well as providing insights from a patient perspective to optimize the communication between physicians and patients during the trial course and by written information. The feedback resulted in minor revisions of words in the patient information sheet to conform with patient concerns.

### Interventions

Treatment will be performed within 14 days after injury. Eligible patients will be randomly allocated to one of two treatment options.

- Surgical treatment
- Non-surgical treatment with the option of early crossover surgery at 6–12 weeks [17] for SHAFT-Y and at 12–26 weeks for SHAFT-E

We anticipate that surgical treatment will include plate osteosynthesis (minimal invasive plate osteosynthesis or open reduction internal fixation), intramedullary nailing (antegrade and retrograde), and external fixation. Plate and nail types, screw configurations, and surgical approaches will be decided by the surgeons. The procedure will be conducted or supervised by a senior consultant.

Non-surgical treatment will include sugar tong splint, plaster splints, hanging casts, or functional bracing such as the Sarmiento brace and will be worn until a surgeon removes it.

All patients will be advised to follow this rehabilitation protocol (Table 1).

**Table 1:** Rehabilitation protocol

Phases (approx. timepoints)	Treatment (mobilization)	Explanation
**Non-surgical**		
Phase 1 Emergency department (ED) (0 weeks)	Apply immobilization device. (Wrist and fingers are recommended to be moved within immobilization device for anti-edema).	Immobilization device should not be taken off (dressing, hygiene). Await decrease of swelling and acute pain
Phase 2 (0–2 weeks)	Shift to brace, if not applied in ED. Physiotherapy can be introduced. (Unrestricted and unloaded active range of motion within the limitations of the brace are allowed).	Brace should always be carried. Patients allowed to lift objects, equivalently to a can of milk (max 1 kg). Physiotherapy can be started to introduce simple movements.
Phase 3a (6 weeks, can be extended to a maximum of 12 weeks)	Fracture is tested gently for instability in patients 18–64 years. If stable, continue to phase 4. If unstable or uncertain stability, return to phase 2 and extend period with brace or consider early crossover surgery.	a. The fracture is not sufficiently healed and needs more time with brace treatment. b. The fracture is grossly unstable and there is a risk of nonunion. Surgical fixation could be beneficial.
Phase 3b (12 weeks, can be extended to a maximum of 26 weeks)	Fracture is tested gently for instability in patients ≥ 65 years. If stable, continue to phase 4. If unstable or uncertain stability, return to phase 2 and extend period with brace or consider early crossover surgery.	a. The fracture is not sufficiently healed and needs more time with brace treatment. b. The fracture is grossly unstable and there is a risk of nonunion. Surgical fixation could be beneficial.
Phase 4 (Patients < 65 years: 6–12 weeks) (Patients > 65 years: 12–26 weeks)	Brace is removed. Continue physiotherapy. (Unrestricted active range of motion of shoulder and elbow with gradual loading).	Fracture is clinically healed. Physiotherapy to regain full range of motion and strength. Movements should be within the threshold of pain.
**Surgical**		
Phase 1 (0 weeks)	Apply immobilization device. (Wrist and fingers are recommended to be moved within immobilization device for anti-edema).	Immobilization device should not be taken off (dressing, hygiene). Await date of surgery.
Phase 2 (0–2 weeks)	Surgical treatment. Physiotherapy can be introduced. (Unrestricted active range of motion, unloaded).	Patients allowed to lift objects, equivalently to a can of milk (max 1 kg). Caution due to wound healing
Phase 3 (6 weeks)	Continue physiotherapy. (Unrestricted active range of motion, starting gradual loading).	Movements should be within the threshold of pain
Phase 4 (7 weeks)	Continue physiotherapy. (No restrictions, active range of motion with full load).	Physiotherapy to regain full range of motion and strength

### Criterias for early crossover

Patients can be offered to undergo early crossover fixation with a surgical procedure of the surgeon’s choice, if one of these criteria are met:

- Unacceptable pain experienced by the patient
- Severe pain with gross instability of the fracture site assessed by:
- Unable to en bloc elevate the arm due to clear fracture instability
- Gentle manipulation of the fracture site. Gentle manipulation should respect the risk of callus breakage
- Severe problems tolerating the brace, e.g., discomfort, skin irritation, wounds, and hygiene problems.

The patients that undergo early crossover surgery will have the reason for crossover thoroughly noted. We anticipate the surgical procedures will be similar to the ones previously mentioned with the possible addition of bone graft.

### Randomization

A computerized database software, Research Electronic Data Capture (REDCap)© [38] will be used to generate an irreversible random allocation sequence and perform block randomization with selected block sizes of 2 and 4, which will be stratified on site and age (18–64 and +65). Patients will be assigned to the trial with an allocation of 1:1 to either surgical treatment or non-surgical treatment. The trial worker acquires the allocated treatment from the central coordinator with randomization rights to REDCap. The trial worker then initiates the treatment, either by scheduling the surgery date or applying the chosen non-surgical method.

### Protocol violation

- Lost to follow-up
- Treatment crossover outside the pre-defined interval for early crossover surgery

Patients that meet any of these criteria will remain in the study and be included in the intention-to-treat analysis but omitted from a per-protocol analysis.

### Participant withdrawal

If the patient withdraws the consent, patients will be included in the statistical analysis through multiple imputation, if baseline data is obtained, otherwise imputation is not possible, and the patient will be replaced to meet the calculated sample size.

### Blinding

The trial will consist of several levels of blinding:

- The primary outcome will be blinded to everyone involved in the trial, apart from the patients and a central trial worker (non-physician), who will only review the questionnaire for completion in REDCap
- The statistical analysis will be conducted by a blinded biostatistician

### Outcome timepoints

Subjective and objective outcome measures will be obtained at the following time points: pre-injury, baseline, 6 weeks, 12 weeks, 26 weeks, 52 weeks, 2 years, and 5 years.

### Baseline data

The following baseline data will be collected after enrollment: age, sex, height, weight, arm dominance, American Society of Anesthesiologists (ASA) grade, pre-injury UCLA activity score [37], list of current diagnosis and medication, mechanism of injury, radiological variables (AO/OTA and fracture location), previous surgery to the arm, tobacco and alcohol habits, employment and educational status. DASH and European Quality of Life – 5 dimensions (EQ-5D-5L) questionnaires will be sent by REDCap immediately after randomization to obtain pre-injury scores.

### Primary outcome measure

The primary outcome is the DASH score at 52 weeks [3]. The DASH will also be assessed at pre-injury, 6, 12 and 26 weeks, and 2 and 5 years. The DASH score is a 30-item self-reporting patient-reported outcome measure specific for physical function and symptoms of the upper limb. Scores range from 0 (no disability) to 100 (most severe disability). The DASH score is validated for the target population [39] and has undergone cross-cultural adaptation in Danish, Swedish and Norwegian [40].

### Secondary outcome measures

Secondary outcomes consist of the DASH score, a self-reported measure of health-related quality of life (EQ-5D-5L), complication rates, visual analog scale (VAS) for pain from the arm, a functional outcome score (Constant-Murley), and anchor questions including clinical anchors, retrospective global transition questions and a binary repeat treatment question. Secondary outcome measures will be assessed at baseline, 6 weeks, 12 weeks, 26 weeks, and 52 weeks (Fig. 3):

- DASH score at earlier timepoints than 52 weeks (MIC [39], 7 and 10 respectively to age 18–64 and 65+)
- General health status questionnaire measured by EQ-5D-5L [41] (MIC [42], 0.074)
- Complications after treatment will be recorded and include local complications, early general complication, and mortality:
- Local complications: Infection (needing antibiotic treatment with or without debridement), nerve or vascular injury, surgical revision (due to implant malpositioning, hardware failure, aseptic loosening, or peri-implant fracture), and tolerance problems with a brace (discomfort resulting in non-compliance of wearing the brace
- Early general complications needing hospitalization within 12 weeks from primary and secondary treatment [43, 44]:
- Major adverse cardiac events (MACE) including myocardial infarction, heart failure, cardiomyopathy and cardiac arrythmias.
- Other major adverse events include all-cause hospitalization other than the defined MACE.
- Mortality
- Pain is assessed by using VAS. Patients are asked to assess their overall pain from the arm from 0 to 100 in a day (MIC [45], 16.55mm)
- Constant-Murley score [46] (MIC [39], 6.1). The subscales of strength and range of motion (ROM) will be depicted. ROM (flexion-extension) of the elbow will be recorded
- Clinical anchor (CA) questions were presented with 5 response options (RO) and analyzed as a 5-point Likert scale:

Q: In general, would you say your health is?

RO: Excellent, Very good, Good, Fair, Poor

*Q: How would you describe the results of the (operation/non-surgical treatment)?*

RO: Excellent, Very good, Good, Fair, Poor

*Q: How would you describe the function of your upper arm?*

RO: Excellent, Very good, Good, Fair, Poor

*Q: How would you describe the pain from your upper arm?*

RO: None, Mild, Moderate, Severe, Extreme

- Retrospective global transition questions (RGTQ) presented with 5 response options and analyzed as a 5-point Likert scale:

*Q: Overall, how would you describe your general health now, compared to after the (operation / non-surgical treatment)?*

RO: Much worse, A little worse, About the same, A little better, Much better

*Q: Overall, how would you describe your upper arm now, compared to after the (operation/non-surgical treatment)?*

RO: Much worse, A little worse, About the same, A little better, Much better

*Q: How would you describe the change in physical function in your upper arm since after the (operation/non-surgical treatment)?*

RO: Much worse, A little worse, About the same, A little better, Much better

*Q: How would you describe the change in pain from your upper arm since after the (operation/non-surgical treatment)?*

RO: Much worse, A little worse, About the same, A little better, Much better

- Binary repeat treatment question (BRT) presented with a binary response option:

*Q: With the knowledge and experience you have gained of the treatment; would you then choose the same treatment again for a similar fracture?*

RO: Yes, No

The DASH, EQ-5D-5L, and CA questions will be sent by email or mail and patients are asked to complete the questionnaires before visits. The questionnaires will be reviewed for missing data by a trial worker and patients will be assisted with completing the questionnaires if any data is missing, without interference from the medical staff. The trial worker collects the missing values before consultation with the physician. The physician then collects the additional outcome measures at the consultation and enters data directly into REDCap.

### Explorative outcome measures

The following explorative outcome measures are collected for research purposes to compare patient groups and treatment. Time points for data collection are depicted in Fig. 3.

- Long-term DASH score at 2 years and 5 years
- Long-term EQ-5D-5L score at 2 years and 5 years
- Radiological measurements [47]
- Gross instability of the fracture site [17]
- Nonunion
- Time of return to work
- Level of activity by the UCLA activity score [48]

### Other outcome measures

Any ancillary outcome measure or analysis will be reported.

## Statistical analysis plan

### Hypothesis

The null hypothesis is:

- The DASH score at 52 weeks after surgical treatment is not superior to non-surgical treatment with the option of early crossover surgery in patients with humeral shaft fractures

### Sample size

The two groups (SHAFT-Y and SHAFT-E) require individual sample size calculations. Two standard deviations (SDs) were obtained from the data of the FISH trial [13] and were separated in age groups of 18–64 years and 65 years and above. By the distribution-based approach, one half a SD corresponds to the minimal important change (MIC) [49]. The calculations are powered to detect a MIC of 7 points in the young and 10 points in the elderly group in DASH, respectively. Two independent means sample size calculations were performed. For SHAFT-Y the following data were included: mean difference = 7.0, SD= 14.91, *α*= 0.05, and power= 0.8. For SHAFT-E the following data were included: mean difference= 10.0, SD= 18.59, α= 0.05 and power= 0.8. Based on the preceding assumptions and including an attrition of 15%, the total sample size is estimated to 163 patients for SHAFT-Y and 124 patients for SHAFT-E.

The FISH trial [13] experienced 5% attrition, since more sites are involved in SHAFT the attrition is set to 15%.

The steering committee can decide to pool data from both RCTs (SHAFT-Y, SHAFT-E) if recruitment is prolonged.

## Statistical methods

The data will be analyzed using computerized statistical software and all data will be entered into REDCap.

### Primary analysis

Descriptive statistics will be used to report demographic data. Demographic data and outcome measures will be tested visually and statistically (i.e., Shapiro Wilks test). Numeric variables will be summarized by means, standard deviations, and 95% confidence intervals (95% CI). Median and interquartile ranges will be used when normal distribution is not met. Categorical variables will be summarized by frequency and proportion. For group comparison with numerical data, a student’s t-test will be used if data is normally distributed, otherwise, a non-parametric test will be used. For categorical data, a chi-square test will be used for group comparison. An intention-to-treat (ITT) analysis of the primary outcome will be conducted by univariable linear regression, including all patients that do not meet the withdrawal criteria, and will be conducted to minimize bias within results. A sensitivity analysis will test the effects of non-adherence to protocol by conducting a per-protocol analysis and includes only patients who comply with the protocol. For missing data points in an outcome measure, a multiple imputation analysis using predictive covariates (age, sex, smoking, alcohol, UCLA activity, ASA grade) [50–52] will be conducted to deal with nonresponse bias. For comparison, we will carry out a sensitivity analysis excluding all the missing values.

Data will be considered statistically significant if *p* values < 0.0471.

### Secondary analysis

In order to validate data, a linear regression analysis will be computed with the DASH score as the dependent variable and treatment modality as the independent variable. Additional regression analysis will be carried out between the early crossover group and the primary treatments. A multivariate regression analysis will be conducted to adjust for potential confounders. Variables adjusted for are: age, sex, smoking, alcohol, UCLA activity, and ASA grade. Furthermore, we will analyze the longitudinal observations by applying a linear mixed-effects regression model, including modality and time as well as a modality-time interaction as fixed effects and a random intercept for each patient. Data will be summarized as coefficients with 95% CIs and variance will be summarized as *r*-squares, adjusted *r*-squares, predicted *r*-squares, and standard errors. Coefficients will be considered statistically significant if *p* values < 0.05.

## Discussion

High-level evidence on the treatment of humeral shaft fracture treatment is sparse, but several RCTs are planned or ongoing [25–28]. The current discussion on surgical treatment versus non-surgical treatment has been ongoing for more than a decade [53]. The discussion has been intensified with recent RCTs showing surprisingly high nonunion rates in the non-surgical groups [13, 23]. The orthopedic research community has demanded the need of more high-quality trials [19, 21]. Thus it is our aim that SHAFT will contribute to the increasing quality of evidence published for the decision-making of treatment of humeral shaft fractures by conducting a pragmatic, two-arm, multicenter, superiority, randomized controlled trial.

The trial is planned pragmatic by introducing real-life treatment courses, to comply with the trial objective of comparing the effectiveness of treatments and furthermore increase external validity.

The distribution of the study population is bimodal, but with a steep increase in incidences from the fifth decade [2, 54]. Our study group received demographic data from the FISH trial [13], which paradoxically, showed a clear difference in the number of recruited patients in the age groups of 18–64 years versus +65 years, favoring the “young” group. To protect the trial against imbalance, age is defined as a stratified variable to ensure equal distribution of age (18–64 and +65) in the treatment groups. The cut-off point of 65 years is decided by the steering committee since it is commonly used in ortho-geriatric research as the cut-off point [55, 56]. Furthermore, Scandinavians can get their retirement pension from around 65 years of age, which will provide us with a more homogenous group considering patient demand of upper extremity function. Moreover, the data from the FISH trial showed a difference in SDs of DASH for patients 18-64 years 14.91 and 18.59 for patients above 65 years. Thus, by the distribution-based approach described by Norman et al. [49], one half a SD corresponds to the MIC, which closely relates to 7 and 10 points used for DASH in humeral shaft fracture literature [39, 57]. Consequently, we have chosen to use 7 and 10 points as MICs, thereby having to recruit 163 young adults (SHAFT-Y) and 124 elderly (SHAFT-E) and to analyze data independently for each group.

Recruitment of 287 humeral shaft fractures presents a challenge in a randomized setting as the annual incidence rate is 14.5 per 100.000 [54]. To overcome this, we will be recruiting from multiple sites across 3 Scandinavian countries with comparable healthcare systems and with cultural and demographic similarities. Despite these overall similarities, surgical management can differ from site to site. To prevent against bias in the surgical group, the randomization will be stratified by the study site. One drawback of this type of stratification can be the risk of open blocks in multiple sites, which can give rise to allocation bias. To limit this the randomization will be conducted in small permuted blocks.

One RCT demonstrated a statistically significant difference in a subgroup analysis between the late surgical crossover and primary surgical treatment after 12 months, favoring early surgery, with respect to possible confounding [13]. The research group has furthermore in a recent study showed the same difference between a late surgical crossover and primary successful treatment after 2 years [15]. To avoid prolonged failed treatment courses, and to minimize overall nonunion rates, the trial has implemented an option of early crossover of non-surgical treatment, if certain criteria for delayed union are met. The criteria for early crossover surgery are identical for each age group but differ regarding the time of crossover. The term “early crossover” and “delayed union” were discussed intensively by the study group. “When has the treatment failed?” Crossover to surgical treatment after non-surgical treatment is not uncommonly performed within 12 weeks for the young and is described due to lack of healing and early non-surgical failure [58, 59]. One can argue that a portion of these patients will unite without intervention [60], but multiple studies have currently shown an association between gross mobility from the fracture site to nonunion in a young population after 6 weeks [17, 50, 60]. For the elderly, our clinical experience is that healing first can be anticipated after 12 weeks. We therefore defined delayed union in SHAFT-Y between 6 and 12 weeks and SHAFT-E between 12 and 26 weeks. Furthermore, endpoints were established to create a clear timepoint of when a treatment has failed, which we believe the orthopedic society needs, since there is no consensus in nonunion for humeral shaft fractures [9, 12, 14, 61].

The DASH questionnaire is adopted by the study group as the primary outcome as it is the best psychometrically validated and cross-culturally adapted PROM for humeral shaft fractures [39, 62]. The full DASH questionnaire is preferred over the QuickDASH by patients through simple semi-structured interviews. The interviews were simple in a sense that the objective was to determine the “most preferred” questionnaire rather than assess the content validity of each questionnaires [63]. The moderator was instructed in the conduct of qualitative interviews by an expert in qualitative research, but not properly trained. This could give challenges with bias, if the moderator preferred one questionnaire. This was sought to be reduced by the semi-structured guide, which was intended to make patients reflect, firstly on their treatment course and secondly on the questionnaires and its relevance, comprehensibility, and comprehensiveness, to induce a thought-through answer to the question: “Which questionnaire do you prefer?”

Through the interviews, it was furthermore recognized that around half of the patients failed to complete one or more questions, but none more than three, which is the cut-off for the score calculation to be valid [3]. To provide a margin of security for missing data, an unveiling trial worker will review the questionnaire for missing answers. Finally, to prevent performance bias, patients will complete the DASH questionnaire prior to visits with the physician, thereby blinding the physician.

The SHAFT trial is a pragmatic multicenter RCT, that will compare the effectiveness between surgical treatment versus non-surgical treatment in humeral shaft fractures, including a variety of fracture morphology, while taking the dilemmas within the population into account by splitting the population by age and providing the orthopedic society with an interval for early cross-over surgery.

### Trial status

Protocol version 3.

Author: DK.

The trial was registered on 05/10/2020 in clinicaltrials.gov (NCT04574336). Last updated 02/08/2022 to conform with protocol updates. https://clinicaltrials.gov/ct2/show/NCT04574336

Recruitment is anticipated to start on 1/3/2022 and is expected to be completed on 1/7 2025. If the sample size of 287 patients has not been meet, the extension of the study period will be discussed by the steering committee.

### Cost-effectiveness

A cost-effectiveness analysis of surgical treatment versus non-surgical treatment with crossover will be undertaken. Utility measures will be based on EQ-5D scores to assess the improvement of each treatment in quality-adjusted life-tear (QALY) over one-year post-treatment. Effectiveness will be assessed by the incremental cost-effectiveness ratio.

## Supplementary Information

**Additional file 1:.** Appendix 1

## Data Availability

Study-related information about patients is secured in REDCap [38]. REDCap is a secure web application with authentication and data logging for managing databases. Access to the database will be limited to trial workers currently working on the trial. Their rights will be limited to data entry only. Principal investigator will keep a list of persons with access. All published data will be fully anonymized of patient identifiers. Furthermore, the trial has been approved by the Danish Data Protection Agency and will conform to the Act on Processing Personal Data. Trial results are planned to be published in an international peer-review journal.

## Abbreviations

SHAFT: Scandinavian Humeral diaphyseal Fracture Trial
SHAFT-Y: Scandinavian Humeral diaphyseal Fracture Trial – Young
SHAFT-E: Scandinavian Humeral diaphyseal Fracture Trial – Elderly
RCT: Randomized controlled trial
WHO: World Health Organization
ISRCTN: International Standard Randomised Controlled Trial Number
SPIRIT: The Standard Protocol Items: Recommendations for Interventional Trials
PRECIS-2: PRagmatic Explanatory Continuum Indicator Summary-2
RNP: Radial nerve palsy
ED: Emergency department
ASA classification: American Society of Anesthesiologists classification
UCLA activity score: University of California, Los Angeles activity score
DMC: Data Monitoring Committee
PROM: Patient-reported outcome measure
PR: Patient representative
REDCap: Research Electronic Data Capture
EQ-5D: European Quality of Life – 5 dimensions
VAS: Visual analog scale
CA: Clinical anchors
RGTQ: Retrospective global transition questions
BRT: Binary repeat treatment
QALY: Quality-adjusted life-year
FISH: Finnish Shaft of the Humerus
ITT: Intention to treat
DASH: Disability of Arm, Shoulder, and Hand
MIC: Minimal important change
SD: Standard deviations
ASA: American Society of Anesthesiologists Score
BMI: Body mass index
ROM: Range of motion
MACE: Major adverse cardiac event
