# Supplementary material for: Surgical versus non-surgical treatment of humeral SHAFT fractures compared by a patient-reported outcome: the Scandinavian Humeral diAphyseal Fracture Trial (SHAFT)—a study protocol for a pragmatic randomized controlled trial
Source: Trials. 2022 Jun 2;23:453. doi: 10.1186/s13063-022-06317-6 (PMC9161482; doi:10.1186/s13063-022-06317-6)
Supplement: Supplementary file 1 — Additional file 1:. Appendix 1 [file 13063_2022_6317_MOESM1_ESM.docx]

**Appendix: PRECIS-2 scores for trial domains**

|  | **Domain** | **Score** | **Rationale** |
| --- | --- | --- | --- |
| 1 | **Eligibility Criteria** | 4 | Rather pragmatic: Patients with a humeral shaft fracture with/or without a fracture extension are included, this fracture morphology is not included in any other studies but seen in a clinical setting. The exclusion criteria are predominately absolute indications for either treatment. |
| 2 | **Recruitment** | 5 | Very pragmatic: No additional efforts are made to recruit patients. They are all referred to our out-clinic follow-up as usual, in which recruitment will take place. |
| 3 | **Setting** | 5 | Very pragmatic: Identical setting to usual care. Out-clinic follow-up in an Orthopedic Department where patients would go for advice and treatment. |
| 4 | **Organization** | 3 | Equally pragmatic/explanatory: Patients are asked to complete two questionnaires prior to consultations. These questionnaires could be implemented to usual care if the results from the questionnaires shows a predictive factor in treatment effect. No need for more staff, but the data collection is more extensive than usual care and some outcomes will need additional devices for measurements. |
| 5 | **Flexibility - Delivery** | 5 | Very pragmatic: No specific protocol for intervention or usual care. Surgeon-dependent procedures. Full flexibility. |
| 6 | **Flexibility - Adherence** | 5 | Very pragmatic: Full adherence. There is no scheduled monitoring for compliance of usual care nor surgical treatment. |
| 7 | **Follow-up** | 2 | Rather explanatory: Patients are followed with questionnaires after 3 weeks, 2 years and 5 years. And further given 2 extra physical follow-up visits at 6 months and 12 months. Visits are longer than usual and more extensive data are collected. |
| 8 | **Primary outcome** | 3 | Equally pragmatic/explanatory: DASH is a surrogate for disability and symptoms. It is highly relevant to investigate the difference between the effect of non-surgical vs surgical treatment. The questions answered in DASH are obviously important for patients, yet the results do not translate directly. |
| 9 | **Primary analysis** | 5 | Very pragmatic: ITT with multiple imputation |
|  | **Sum total** | 37 | Pragmatic trial. The average PRECIS-2 score is 4.11. Since the average is >3 points, it can be recognized as a pragmatic trial(1) |
